# An Association between HTRA1 and TGF-β_2_ in the Vitreous Humor of Patients with Chorioretinal Vascular Diseases

**DOI:** 10.3390/jcm13175073

**Published:** 2024-08-27

**Authors:** Yoko Fukushima, Shizuka Takahashi, Machiko Nakamura, Tatsuya Inoue, Yusuke Fujieda, Toshiyuki Sato, Shingo Noguchi, Motokazu Tsujikawa, Hirokazu Sakaguchi, Kohji Nishida

**Affiliations:** 1 Department of Ophthalmology, Osaka University Graduate School of Medicine, Suita 565-0871, Osaka, Japan; youko.fukushima@ophthal.med.osaka-u.ac.jp (Y.F.); shizuka.yoshihara@gmail.com (S.T.); sakaguh@gmail.com (H.S.); 2 Integrated Frontier Research for Medical Science Division, Institute for Open and Transdisciplinary Research Initiatives (OTRI), Suita 565-0871, Osaka, Japan; 3 Department of Ophthalmology, Higashiosaka City Medical Center, Higashiosaka 578-8588, Osaka, Japan; 4 Daiichi Sankyo Co., Ltd., Chuo-ku 140-8170, Tokyo, Japan; nakamura.machiko.jb@daiichisankyo.co.jp (M.N.); inoue.tatsuya.cg@daiichisankyo.co.jp (T.I.); fujieda.yusuke.fg@daiichisankyo.co.jp (Y.F.); shingo.noguchi.ni@daiichisankyo.co.jp (S.N.); 5 Department of Biomedical Informatics, Osaka University Graduate School of Medicine, Suita 565-0871, Osaka, Japan; moto@ophthal.med.osaka-u.ac.jp; 6 Department of Ophthalmology, Gifu University Graduate School of Medicine, Gifu 501-1194, Gifu, Japan

**Keywords:** HTRA1, TGF-β, chorioretinal vascular disease

## Abstract

**Background**: The aim of this paper was to investigate the protein concentrations of high-temperature requirement A 1 (HTRA1) and transforming growth factor-β (TGF-β) in the vitreous humor of patients with chorioretinal vascular diseases. **Methods**: This study measured protein concentrations of HTRA1, TGF-$\beta_{1-3}$, and vascular endothelial growth factor A (hereinafter called VEGF) in the vitreous humor from seven eyes of patients with chorioretinal vascular diseases (age-related macular degeneration, diabetic macular edema, and retinal vein occlusion) and six control eyes (idiopathic epiretinal membrane and macular hole). We analyzed the mutual relationship among the protein levels. **Results**: The protein levels of HTRA1 and VEGF were significantly increased in the chorioretinal vascular disease group compared with the control group (1.57 ± 0.79 $\times \;10^{-9}$ mol/mL vs. 0.68 ± 0.79 $\times \;10^{-9}$ mol/mL, *p* = 0.039; 3447.00 ± 3423.47 pg/mL vs. 35.33 ± 79.01 pg/mL, *p* = 0.046, respectively). TGF-$\beta_{2}$ levels were not significantly different between groups (2222.71 ± 1151.25 pg/mL for the chorioretinal vascular disease group vs. 1918.83 ± 744.01 pg/mL for the control group, *p* = 0.62). The concentration of HTRA1 was strongly associated with TGF-$\beta_{2}$ levels in the vitreous humor, independent of VEGF (r = 0.80, *p* = 0.0010). **Conclusions**: We revealed that vitreous HTRA1 was increased in patients with chorioretinal vascular diseases and strongly correlated with TGF-$\beta_{2}$.

## 1. Introduction

Human high-temperature requirement A 1 (HTRA1) is a protease with important roles in various cellular pathological processes [1,2]. The vascular system is a target in diseases related to the central nervous system such as hereditary ischemic cerebral small-vessel disease and age-related macular degeneration (AMD). In patients with hereditary ischemic cerebral small-vessel disease, mutated HTRA1 loses its function as a protease, resulting in vascular obstruction and micro-bleeding due to the aggregation of its substrates and deposits in cerebral vascular walls [3]. In contrast to HTRA1 dysfunction in hereditary ischemic cerebral small-vessel disease, HTRA1 is increased in neovascular AMD while its function is preserved [4,5,6,7]. A mutation in the promoter region of the *HTRA1* gene increases its expression, which is thought to induce pathological angiogenesis [8].

HTRA1 modulates transforming growth factor beta (TGF-β) signaling, which regulates angiogenesis under physiological and pathological conditions [9,10,11,12,13]. All three isoforms, TGF-$\beta_{1}$, TGF-$\beta_{2}$, and TGF-$\beta_{3}$, directly bind to serine/threonine protein kinase type I and type II receptors on the cell membrane surface to transmit their intracellular signals [14,15]. Of the three isoforms, TGF-$\beta_{1}$ promotes sprouting angiogenesis during the vascular development of the brain and retina in mice [13]. In addition, endothelial- and pericyte-derived TGF-β signaling contributed to the formation and maturation of the blood–brain barrier, respectively [16]. Thus, TGF-β signaling regulates the development and homeostasis of blood vessels in the central nervous system. It has been also suggested that TGF-β signaling is a major factor that drives fibrosis in various tissues, as well as angiogenesis [17,18]. TGF-β signaling induces fibrosis via the activation of myofibroblasts, excessive production of extracellular matrix, and inhibition of extracellular matrix degradation [14]. HTRA1-mediated proteolysis is thought to interfere with these functions of TGF-β signaling [2].

In cases of human chorioretinal vascular diseases, TGF-β signaling promotes pro-angiogenic, anti-angiogenic, or pro-fibrotic effects, depending on the disease phase. Several studies have reported higher TGF-β1 levels in neovascular AMD and diabetic retinopathy (DR) patients compared with controls [19,20,21,22,23]. In AMD, TGF-$\beta_{1}$ remained elevated after anti-vascular endothelial growth factor (VEGF) treatment, suggesting the regulation of TGF-$\beta_{1}$ signaling was VEGF-independent [20]. In contrast, recent findings indicated reduced active TGF-$\beta_{2}$ in neovascular AMD [18]. Although aqueous HTRA1 is increased in AMD, its interaction with TGF-β isoforms in chorioretinal vascular diseases is unclear. This study examined the association between HTRA1 and TGF-β under conditions of pathological angiogenesis, vascular homeostasis failure, or potential fibrosis. We measured the protein concentrations of HTRA1, the three isoforms of TGF-β, and VEGF-A (hereinafter called VEGF) in human vitreous humor from patients with chorioretinal vascular diseases, including AMD, DR, and retinal vein occlusion (RVO). In addition, we evaluated correlations between HTRA1, TGF-β, and VEGF.

## 2. Materials and Methods

### 2.1. Study Design

This prospective pilot study was conducted at Osaka University Hospital between October 2017 and March 2021.

### 2.2. Participants

This study consisted of all consecutive patients who underwent intravitreal injection, senile cataract surgery, or vitrectomy between October 2017 and March 2021 at the Department of Ophthalmology, Osaka University. Patients with neovascular AMD, any stage of DR, and RVO were enrolled into the chorioretinal vascular diseases group. Patients who received anti-VEGF injections within 1 year were excluded from this study. Patients with idiopathic macular hole and idiopathic epiretinal membrane were enrolled into the control group if they did not have a history of diabetes mellitus or chorioretinal vascular disease. We excluded cases with any of the following: insufficient vitreous sample volume or withdrawal of consent.

### 2.3. Ophthalmic Examinations

All patients underwent ophthalmic examinations at baseline, including best-corrected visual acuity measurement, indirect ophthalmoscopy, and fundus photography. The control patients were diagnosed by optical coherence tomography (OCT). Patients with chorioretinal vascular diseases were diagnosed with OCT, fluorescein angiography (FA), and/or indocyanine green angiography.

### 2.4. Collection of Vitreous Humor

Before starting intravitreal injection or vitrectomy, undiluted samples of the vitreous (100 mL) were aspirated using a 30-gauge needle as previously described [24]. A single surgeon (H.S.) collected the samples during vitrectomy and performed intravitreal injections. The samples were aliquoted into sterile tubes for single use and stored frozen at −80 °C.

### 2.5. Measurement of TGF-$\beta_{1}$, TGF-$\beta_{2}$, TGF-$\beta_{3}$ and VEGF in the Vitreous Humor

We measured the levels of HTRA1, TGF-$\beta_{1}$, TGF-$\beta_{2}$, TGF-$\beta_{3}$, and VEGF using the Bio-Plex suspension assay system (except HTRA1) (Bio-Plex 200, BioRad, Hercules, CA, USA) with a commercially available TGF-β Magnetic Bead 3 Plex Kit (Merck Millipore, Burlington, MA, USA) and Human Angiogenesis/Growth Factor Magnetic Bead Panel 1 (Merck Millipore, Burlington, MA, USA). TGF-$\beta_{1}$, TGF-$\beta_{2}$, and TGF-$\beta_{3}$ were measured as total proteins (active and inactive forms) after an acidification process following the kit instructions. Human vitreous was diluted with distilled water 10 times, and the mean value of duplicate measurements was determined. Only values beyond the limit of detection obtained from distilled water were analyzed; otherwise, they were considered not determined and shown as zero in the figures.

### 2.6. Measurement of HTRA1 in the Vitreous Humor

Since the amount of sample collected was insufficient for measurement by multiplex ELISA for HTRA1, we used the more sensitive nano-LC/MS system. For HTRA1 measurements, 3 μL of sample was diluted to 10 μL with H_2_O. Then, the sample was reduced by diluting it with 10 μL of urea solution (8 M urea, 10 mM ethylenediaminetetraacetic acid, 1 M Tris-HCl [pH 8.0]) and 2.5 μL of 20 mg/mL dithiothreitol for 1 h at 37 °C. Then, the sample was alkylated with 2.5 μL of 50 mg/mL iodoacetamide for 1 h in the dark at room temperature. Protein in the sample was digested with trypsin solution (45.8 μL of 1 M Tris-HCl [pH 8.0]; 6 μL of 0.2 mg/mL trypsin/Lys-C mix [Promega, Tokyo, Japan]; 3.2 μL of 1% Protease MAX surfactant, [Promega]) at 37 °C overnight. To terminate digestion, an equal volume of 1% trifluoroacetate was added to the digested sample. Prior to nano-LC/MC, internal standard peptides consisting of chemically synthesized peptides (amino acid sequences: VTAGISFAIPSDK and VTAGISFAIP*SDK [P*: ^13^C_5_, ^15^N_1_-proline]) obtained from Bio-Synthesis Inc. were diluted in 50% acetonitrile to form a 1 nM standard peptide solution. After adding 10 μL of the internal standard peptide solution, the sample was desalted using GL-Tip SDB (GL Science, Tokyo, Japan) and GL-Tip GC (GL Science). Subsequently, the sample was evaporated and resuspended with 0.1% formic acid before nano-LC/MS analysis.

LC/MS analysis was performed using an EASY-nLC 1000 (Thermo Fisher Scientific, Waltham, MA, USA) equipped with Triple TOF 6600 (SCIEX, Tokyo, Japan). An aliquot of 4 μL of protein from each sample was loaded onto a 75 μm × 20 mm Acclaim PePMap precolumn (Thermo Fisher Scientific) and washed with 100% purified water and 0.1% formic acid at a flow rate of 300 nL/min. The precolumn was connected to a 75 μm × 150 mm Nano HPLC Capillary Column (Nikkyo Technos, Tokyo, Japan). The samples were subjected to LC/MS analysis using a 35-min gradient from 5% to 100% buffer B (100% acetonitrile, 0.1% formic acid). To create a calibration curve, standard peptides of different concentrations were quantified. The amount of endogenous HTRA1 in undiluted vitreous humor samples was calculated based on the calibration curve.

### 2.7. Statistical Analysis

Statistical analysis was performed using R software version 4.1.2 (R Foundation for Statistical Computing, Vienna, Austria) [25]. The results are expressed as the mean ± standard deviation. Differences between groups were assessed by Student’s *t*-test (continuous variables) or χ^2^ test (categorical variables). To assess the correlation between HTRA1, TGF-$\beta_{2}$, and VEGF, Spearman’s correlation coefficient with 95% confidence intervals was calculated. *p*-values less than 0.05 were considered to indicate statistical significance.

## 3. Results

Of the 23 eyes from 23 patients enrolled in this study, 10 were excluded for insufficient sample volume (n = 9) and withdrawal of consent (n = 1). This study consisted of six control patients and seven patients with chorioretinal vascular diseases, of whom three had neovascular AMD, two had non-proliferative DR (non-PDR) with diabetic macular edema (DME), one had branch RVO (BRVO) with macular edema, and one had BRVO complicated with DR. One DME case in this study had received an anti-VEGF injection 18 months previously, and the other cases were treatment-naïve. The clinical characteristics of the two groups are summarized in Table 1. There were no significant differences in age, sex, and disease duration between the two groups.

**Figure 1:**
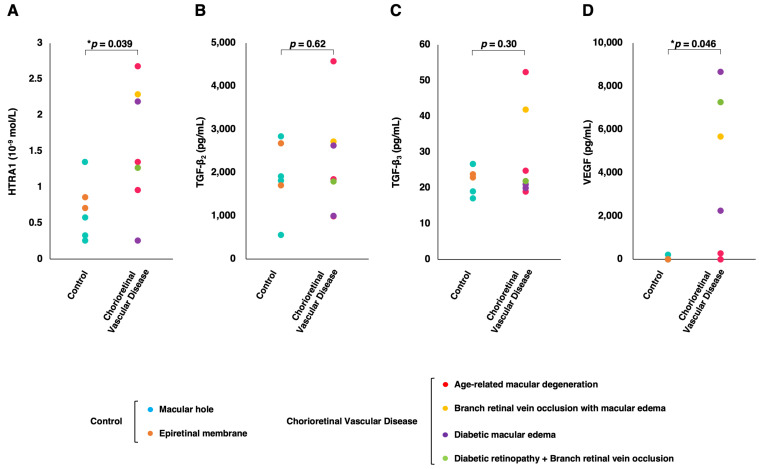
The distribution of the individual vitreous concentrations of (**A**) HTRA1, (**B**) TGF-$\beta_{2}$, (**C**) TGF-$\beta_{3}$, and (**D**) VEGF by group. Note that HTRA1 levels were measured as $10^{-9}$ mol/L, and TGF-$\beta_{2}$, TGF-$\beta_{3}$, and VEGF were measured as pg/mL. *p*-values are indicated in the plots, * *p* < 0.05.

Figure 1 shows the vitreous concentrations of HTRA1, TGF-$\beta_{2}$, TGF-$\beta_{3}$, and VEGF in the control group and the chorioretinal vascular disease group. The mean concentrations of HTRA1 and VEGF were significantly higher in the chorioretinal vascular disease group than in the control group (1.57 ± 0.79 $\times \;10^{-9}$ mol/mL vs. 0.68 ± 0.36 $\times \;10^{-9}$ mol/mL, *p* = 0.039; 3447.00 ± 3423.47 pg/mL vs. 35.33 ± 79.01 pg/mL, *p* = 0.046, respectively). VEGF was not detected in the five control patients. Of the three TGF-β isoforms, TGF-$\beta_{1}$ was below the detection limit in all patients except the patient with BRVO. However, the mean concentrations of TGF-$\beta_{2}$ and TGF-$\beta_{3}$ were not significantly different between the groups (2222.71 ± 1151.25 pg/mL for the chorioretinal vascular disease group vs. 1918.83 ± 744.01 pg/mL for the control group, *p* = 0.62; 28.71 ± 12.11 pg/mL for the chorioretinal vascular disease group vs. 22.70 ± 3.61 pg/mL for the control group, *p* = 0.30, respectively).

**Figure 2:**
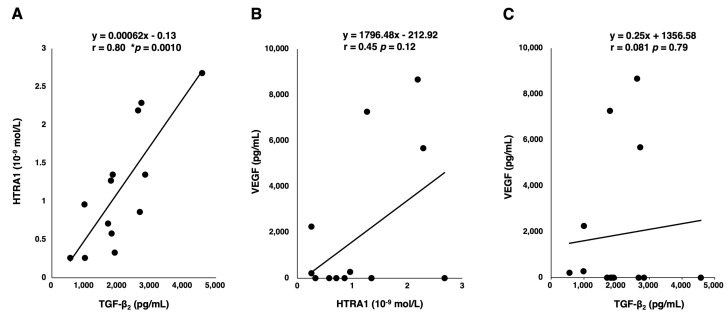
Scatter plots and linear regression lines of the concentrations of (**A**) TGF-$\beta_{2}$ and HTRA1, (**B**) HTRA1 and VEGF, and (**C**) VEGF and TGF-$\beta_{2}$. The equation, correlation coefficient, and statistical significance are provided in the plots, * *p* < 0.05.

In the vitreous humor, the concentration of TGF-$\beta_{2}$ is dominant and is more than 100 times higher than that of TGF-$\beta_{3}$. We assessed the association between TGF-$\beta_{2}$, which is the predominant isoform in the eye, and VEGF/HTRA1. We found that while HTRA1 had a fairly strong correlation with TGF-$\beta_{2}$ (r = 0.80; 95% CI, 0.45–0.94; *p* = 0.0010), HTRA1 and TGF-$\beta_{2}$ were not significantly correlated with VEGF (r = 0.46; 95% CI, −0.13–0.81; *p* = 0.11; r = 0.081; 95% CI, −0.49–0.61; *p* = 0.79, respectively) as VEGF was not detectable in most control patients (Figure 2).

**Figure 3:**
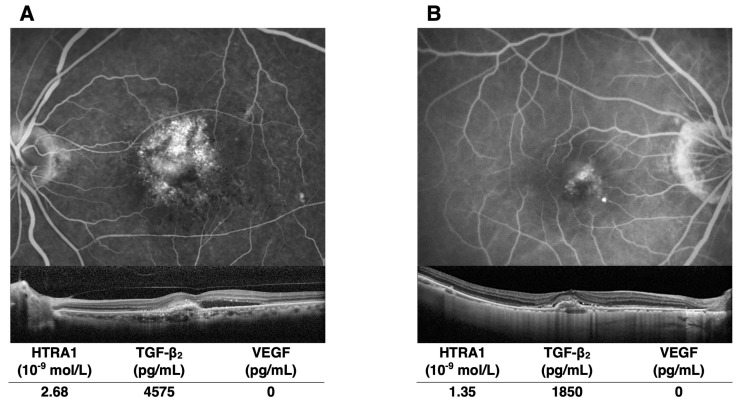

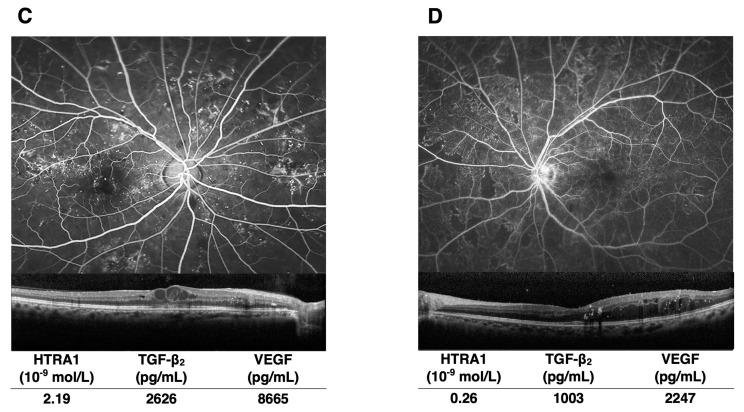
Representative images obtained from patients with AMD and DME. The levels of HTRA1 and TGF-$\beta_{2}$ are elevated in the eyes of patients with more severe hyperpermeability. The HTRA1, TGF-$\beta_{2}$, and VEGF concentrations in the eyes are given below the images. FA in AMD cases (**A**,**B**) was obtained by SPECTRALIS^®^ HRA (Heidelberg Engineering, Heidelberg, Germany), and in DME cases (**C**,**D**) by Optos^®^ (Optos, Marlborough, MA, USA). OCT was obtained by SPECTRALIS for all disease types.

Figure 3 shows four representative cases: two patients with neovascular AMD and two patients with DME. Of the two AMD patients, one with a higher concentration of HTRA1 and TGF-$\beta_{2}$ had larger choroidal neovascularization and more subretinal fluid. Similarly, FA images demonstrated increased vascular leakage from microaneurysms in the DME patient with higher HTRA1 and TGF-$\beta_{2}$ levels. The VEGF concentrations were not related to the extent of vascular hyperpermeability in AMD and DME.

## 4. Discussion

Our study demonstrated that among the vitreous factors measured (HTRA1, TGF-$\beta_{1}$, TGF-$\beta_{2}$, TGF-$\beta_{3}$, and VEGF), higher levels of HTRA1 and VEGF were present in chorioretinal vascular disease patients compared with controls. Although previous studies reported a high concentration of HTRA1 in AMD with choroidal neovascularization [4,5], this study revealed elevated levels of HTRA1 in chorioretinal vascular diseases, including DME and RVO, as well as neovascular AMD. Consistent with previous reports [17,19,26,27,28,29], of the three isoforms of TGF-β, TGF-$\beta_{2}$ was quantitatively predominant in the eyes in this study.

Additionally, we discovered a strong correlation between HTRA1 and TGF-$\beta_{2}$, although neither was correlated with VEGF. Despite a recent study concluding there was no correlation between HTRA1, TGF-$\beta_{1}$, and VEGF in chorioretinal vascular diseases [20], our study demonstrated an association between HTRA1 and TGF-$\beta_{2}$. This discrepancy may be attributed to the differences in the detection limits of the measurement methods. Generally, the LC/MS method is more sensitive than the ELISA method. Indeed, when converted from $10^{-9}$ mol/L to ng/mL, HTRA1 concentrations were 33.4 ng/mL in control patients and 80.7 ng/mL in patients with chorioretinal vascular diseases, which exceeded those previously reported [20]. Another contributing factor might be the differences between vitreous and aqueous humors. Given that we found no correlation between the aqueous HTRA1 concentration and vitreous HTRA1 concentration, we decided to focus on measuring vitreous HTRA1 concentrations. Furthermore, HTRA1 protein may experience a diffusion gradient or protein instability in the human vitreous humor. Therefore, our study is the first to demonstrate a correlation between HTRA1 and TGF-$\beta_{2}$ in humans.

In our study, eyes with higher HTRA1 and TGF-$\beta_{2}$ levels seemed to have stronger vascular permeability by FA, suggesting vascular barrier dysfunction. TGF-β signaling maintains the blood–retina barrier, the integrity of tight and adherens junctions between endothelial cells in the mouse retina under physiological conditions [16]. On the other hand, glia-derived TGF-β damages cellular junctions in cultured endothelial cells [30,31]. Given the increase in TGF-β levels in the vitreous humor of PDR patients [19,28,32], TGF-β signaling might differentially regulate vascular permeability in a context-dependent manner. Elevated TGF-$\beta_{2}$ may be attributed to the breakdown of vascular barriers in progressing chorioretinal vascular diseases. Additionally, endothelial-to-mesenchymal transitions promoted by TGF-β signaling may advance fibrosis and exacerbate vascular permeability by disrupting cellular adhesion [14,17]. Previous studies reported that the protein levels of TGF-$\beta_{2}$ were associated with the progression of retinal fibrosis [33,34,35]. Furthermore, HTRA1 deteriorated pathological conditions by inducing apoptosis in photoreceptor cells [6]. Although which of HTRA1 and TGF-$\beta_{2}$ is upstream of the signaling axis remains unclear, the HTRA1- and TGF-b2-related signaling cascades could lead to advanced chorioretinal vascular diseases.

There were several limitations in this study. Firstly, due to the small sample size, we were unable to examine the protein levels across different diseases. Thus, our results need to be validated in a larger sample size. Nonetheless, as a pilot study, the findings should help enhance our understanding of the pathology of chorioretinal vascular diseases, particularly the finding that there is a strong correlation between HTRA1 and TGF-$\beta_{2}$. Secondly, the results of this study suggest an association between vascular leakage observed by FA and HTRA1 concentrations in the vitreous humor or between affected regions shown by FA and HTRA1 concentrations. A larger sample size would allow for a more comprehensive comparison between clinical findings and protein levels. Thirdly, we used different methods to measure HTRA1 and two other proteins. Because the specificity and affinity of antibodies have an impact on ELISA signals, the protein concentrations of VEGF and TGF-β in the vitreous humor might have been higher if measured using LC-MS. Fourthly, we did not quantify active TGF-β isoforms. A recent study demonstrated a decrease in the active form of TGF-$\beta_{2}$ in the aqueous humor of patients with neovascular AMD [18]. A further investigation will be needed to determine whether the active form of TGF-$\beta_{2}$ also increases in response to elevated total protein levels, or whether decreased active TGF-$\beta_{2}$ in the vitreous humor is a result of compensatory cleavage by upregulated HTRA1, ultimately leading to the regulation of TGF-$\beta_{2}$ signaling. Finally, the degree of vascular leakage could not be quantified due to the use of different imaging devices. The study required FA and OCT imaging for diagnosis at patient enrollment. However, there were no restrictions on the type of equipment used. In future studies, we plan to use specific models for each disease to allow for a quantitative evaluation.

## 5. Conclusions

Vitreous HTRA1 was elevated in patients with chorioretinal vascular diseases and strongly correlated with TGF-$\beta_{2}$ in the human vitreous humor. Because of the lack of an association with VEGF, HTRA1 and TGF-$\beta_{2}$ might be used as biomarkers of vascular dysfunction and predictors of patients with chorioretinal vascular diseases refractory to anti-VEGF therapy. If the involvement of HTRA1 and TGF-$\beta_{2}$ in disease progression is clarified in the future, they may also serve as therapeutic targets.

## Figures and Tables

**Table 1:** Clinical characteristics of the patients in this study.

Characteristics	Control (n = 6)	Chorioretinal Vascular Disease (n = 7)	*p* Value
Male Gender no. (%)	4 (66.67%)	4 (57.14%)	0.93
Age (years); mean ± SD ^1^	67.17 ± 2.79	71.57 ± 10.91	0.40
Disease Duration (mo); mean ± SD ^1^	11.50 ± 14.56	8.43 ± 10.91	0.70

^1^ SD, standard deviation.

## Data Availability

The raw data supporting the conclusions of this article will be made available by the authors on request.

## Abbreviations

The following abbreviations are used in this manuscript:

HTRA1	Human high-temperature requirement A 1
AMD	Age-related macular degeneration
TGF-β	Transforming growth factor beta
VEGF	Vascular endothelial growth factor
DR	Diabetic retinopathy
RVO	Optical coherence tomography
FA	Fluorescein angiography
non-PDR	Non-proliferative diabetic retinopathy
DME	Diabetic macular edema
BRVO	Branch retinal vein occlusion
CI	Confidence interval
